# Macrophages Release Extracellular Vesicles of Different Properties and Composition Following Exposure to Nanoparticles

**DOI:** 10.3390/ijms24010260

**Published:** 2022-12-23

**Authors:** Sarah Deville, Hector Garcia Romeu, Eline Oeyen, Inge Mertens, Inge Nelissen, Anna Salvati

**Affiliations:** 1 Department of Nanomedicine & Drug Targeting, Groningen Research Institute of Pharmacy, University of Groningen, A. Deusinglaan 1, 9713 AV Groningen, The Netherlands; 2 Health Unit, Flemish Institute for Technological Research, Boeretang 200, 2400 Mol, Belgium; 3 Biomedical Research Institute, Hasselt University, Agoralaan Building C, 3590 Diepenbeek, Belgium; 4 Centre for Proteomics (CfP), University of Antwerp, Groenenborgerlaan 171, 2020 Antwerp, Belgium

**Keywords:** extracellular vesicles, nanosafety, alveolar macrophages, high sensitivity flow cytometry

## Abstract

Extracellular vesicles are membrane-bound carriers with complex cargoes, which play a major role in intercellular communication, for instance, in the context of the immune response. Macrophages are known to release extracellular vesicles in response to different stimuli, and changes in their size, number, and composition may provide important insights into the responses induced. Macrophages are also known to be highly efficient in clearing nanoparticles, when in contact with them, and in triggering the immune system. However, little is known about how the nature and composition of the vesicles released by these cells may vary upon nanoparticle exposure. In order to study this, in this work, alveolar-like macrophages were exposed to a panel of nanoparticles with varying surface and composition, including amino-modified and carboxylated polystyrene and plain silica. We previously showed that these nanoparticles induced very different responses in these cells. Here, experimental conditions were carefully tuned in order to separate the extracellular vesicles released by the macrophages several hours after exposure to sub-toxic concentrations of the same nanoparticles. After separation, different methods, including high-sensitivity flow cytometry, TEM imaging, Western blotting, and nanoparticle tracking analysis, were combined in order to characterize the extracellular vesicles. Finally, proteomics was used to determine their composition and how it varied upon exposure to the different nanoparticles. Our results show that depending on the nanoparticles’ properties. The macrophages produced extracellular vesicles of varying number, size, and protein composition. This indicates that macrophages release specific signals in response to nanoparticles and overall suggests that extracellular vesicles can reflect subtle responses to nanoparticles and nanoparticle impact on intercellular communication.

## 1. Introduction

Extracellular vesicles (EVs) are naturally occurring vesicles with different cargoes. They are released by most biological cells both under physiological conditions and under stress [1] and can include many different types of molecules, including specific proteins, lipids, and nucleic acids [2,3,4,5]. EV profiles can vary depending on the metabolic state, activation status, and type of cells, among other factors [6,7]. EVs are key players in intercellular communication, and this makes them promising tools as circulating biomarkers for disease diagnosis and prognosis, but also attractive for drug delivery approaches and therapeutic applications [8,9,10,11,12]. Depending on their content, EVs can modulate a variety of cellular functions and contribute to physiological homeostasis. Hence, the release of EVs can play a role in cellular migration and invasion [13,14] and in immune responses [5].

In recent years, the development of nanoparticles for precision medicine has attracted increasing interest. Lipid-based, polymeric, and inorganic nanoparticles are engineered to overcome biological barriers, prolong circulation times, and deliver therapeutic cargos into specific target tissues, cells, and organelles. Engineering the physicochemical parameters, including size, shape, surface chemistry, and charge, can have a tremendous impact on cellular uptake and distribution and, thereby, in vivo performance of the nanoparticles [15]. Their properties also influence their clearance from the circulation resulting from interaction with the mononuclear phagocytic system (MPS), which is represented by macrophages, neutrophils, and dendritic cells, which take up the nanoparticles and accumulate in the liver and spleen. These phagocytic cells seem to have an increasing preference for particles with increasing sizes above 0.5 µm [16,17]. In contrast, triangular and rod-shaped nanoparticles show more uptake than star-shaped or spherical nanoparticles [18,19]. In terms of surface charge, cationic nanoparticles are generally most rapidly cleared, followed by anionic nanoparticles, whereas neutral or slightly negative nanoparticles have the longest half-lives in circulation. Besides clearance, interactions with the MPS can cause toxicity, as these cells trigger the immune system involving the secretion of reactive oxygen species, pro-inflammatory cytokines, and induction of antigen presentation. The type and magnitude of immune response to nanoparticles are greatly affected by the particles’ size, shape, and surface properties [20]. When triggered by pathogens (e.g., bacteria or viruses), which can be considered biogenic particles, phagocytes have also been observed to release EVs with specific cargo that regulates the innate and adaptive immunity in the recipient cells [21]. However, following engineered nanoparticles’ interactions with the MPS, little is known about the effects on the production, composition, and role of the released EVs.

Exposure to nanoparticles and subsequent nanoparticle uptake and accumulation inside cells can potentially alter the release of EVs [22]. Additionally, EVs and nanoparticles partly traffic in similar compartments. Thus, nanoparticle accumulation may affect EV biogenesis, composition, and secretion [23,24]. Only a few studies have addressed this question and reported altered EV release and function in cells exposed to nanoparticles [25,26,27,28].

In order to study this question, here we have characterized the EVs produced by macrophages, selected as one of the very first cells in contact with nanoparticles following exposure. Murine Max Planck Institute (MPI) macrophages were used as a model of non-transformed primary alveolar-like macrophages [29]. These are granulocyte–macrophage colony-stimulating factor (GM-CSF) derived cells, which maintain key features of primary macrophages while maintaining a relatively high proliferation rate [29]. The MPI cells were exposed to a panel of nanoparticles having the same size and shape, but different surface and composition and known to have a very different impact on cells. These included 50 nm positively charged amino-modified polystyrene (NH_2_-PS) known to induce cell death on many cells [30,31], 50 nm negatively-charged carboxylated polystyrene (COOH-PS), usually well tolerated, and 50 nm plain silica (SiO_2_) nanoparticles, for which in some cases toxic responses were observed [32,33]. We previously characterized in detail the impact of these nanoparticles on MPI cells and showed that both NH_2_-PS and SiO_2_ induce cell death, but via very different mechanisms [34]. More specifically, while the COOH-PS nanoparticles were well tolerated, the NH_2_-PS nanoparticles induced a peculiar mitochondrial membrane hyperpolarization, accompanied by endoplasmic reticulum stress and an increase of reactive oxygen species (ROS), ultimately leading to cell death. In contrast, exposure to SiO_2_ nanoparticles induced ROS production, loss of mitochondrial membrane potential and cell death by apoptosis [34]. Thus, the selected panel of nanoparticles was used to determine potential differences in the proteome content of the EVs released by the macrophages, depending on the type of nanoparticles and their different impact on these cells. Additionally, care was taken to optimize the experimental conditions in order to expose the cells to these nanoparticles at the sub-toxic concentration for a few hours and then collect the released EVs several hours after exposure. This allowed us, on the one hand, to avoid confusing EVs with the release of debris or apoptotic bodies when too strong toxic conditions are applied, and on the other also to focus on changes in EV release as a novel way to detect and characterize more subtle responses induced by sub-toxic exposure to nanoparticles.

Other studies reported changes in proteomic signatures of macrophages upon exposure to amorphous silica nanoparticles at sub-toxic concentrations, including changes in the release of NO and pro-inflammatory cytokines, but without information on potential changes in the secreted EVs [35]. Changes in released EVs were instead characterized in more detail for macrophages exposed to dust silica in the context of silicosis [36]. It was found that, indeed, the released EVs differed in composition and affected intercellular communication, and more specifically, they induced fibroblast transdifferentiation into myofibroblasts [36]. Overall, the analysis of the composition of the EVs produced by cells may provide novel and important insights into the mechanisms of cellular responses to nanoparticles.

## 2. Results and Discussion

### 2.1. Optimization of In Vitro Nanoparticle Exposure Conditions

As a first step, nanoparticle size and zeta potential were determined after dispersion in relevant media (Supplementary Table S1). Dispersion in a complete cell culture medium with serum and GM-CSF supplementation led to an increase in nanoparticle size and a decrease in zeta potential absolute value, closer to neutrality, as expected upon corona formation [30]. All nanoparticle dispersions remained stable even after 24 h exposure in the conditions used for experiments with cells (Supplementary Table S1).

**Figure 1:**
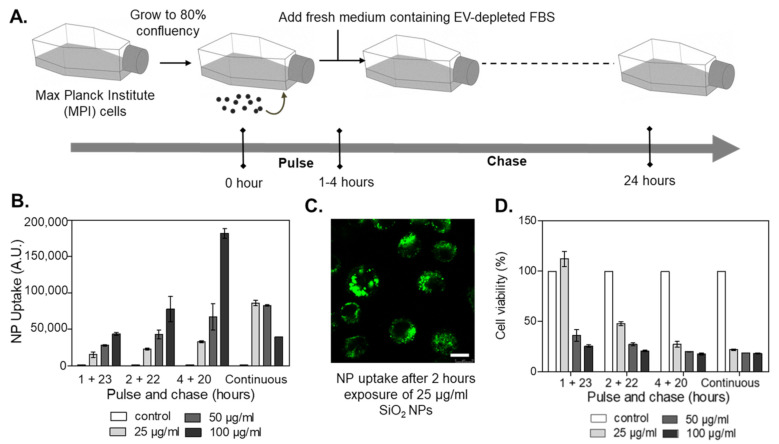
Optimization of pulse-chase nanoparticle exposure conditions in MPI cells. (**A**) Cells were exposed (“pulse”) at different times to nanoparticles, and thereafter, the medium was replaced by fresh medium containing EV-depleted FBS (“chase”). After 24 h, the cells and the corresponding conditioned cell medium were collected for further downstream analyses. (**B**) Nanoparticle uptake by MPI cells exposed to different doses of fluorescently labeled SiO_2_ nanoparticles under continuous exposure or different pulse and chase conditions. The results are the mean ± standard deviation obtained by flow cytometry from 2 replicate samples per condition. (**C**) Images obtained by confocal fluorescence microscopy of MPI cells exposed for 2 h to 25 µg/mL fluorescently labeled SiO_2_ nanoparticles. The results confirmed very efficient nanoparticle uptake by MPI cells. Scale bar: 10 µm. (**D**) Cell viability obtained by MTT after exposure to SiO_2_ nanoparticles. The results are the mean ± standard deviation from 3 replicate samples per condition, normalized by the results obtained in untreated control cells.

We then optimized exposure conditions to avoid strong cytotoxic responses, characterized by the generation of cell debris and apoptotic bodies, which could interfere with EV separation and characterization. We have previously characterized in detail the response of the MPI cells to the selected nanoparticles [34]. Based on these observations, MPI cells were exposed to sub-toxic concentrations of nanoparticles for 1 to 4 h (“pulse”), followed by long-term incubation up to 24 h in fresh, nanoparticle-free media supplemented with EV-depleted FBS (“chase”) (Figure 1A) (We note that for the selected nanoparticles no strong evidence of degradation or ion release has been reported within this time frame). Uptake and cell viability measurements were used to select exposure conditions that guaranteed substantial nanoparticle uptake without evident toxicity (Figure 1B–D). Thus, the optimized conditions were set at 1 h pulse exposure to 25 µg/mL nanoparticles, followed by extracellular nanoparticle removal and 23 h chase in an EV-depleted medium. Exposure to 100 ng/mL lipopolysaccharide (LPS) under the same conditions was used as a positive control for the induction of an inflammatory response. In the selected conditions, minimal impact on metabolic activity and no cell death were detected (Supplementary Figures S1 and S2). Additionally, flow cytometry analysis of mannose receptor CD206 and the major histocompatibility complex II (MHCII) showed that, as expected, exposure to LPS decreased the number of the so-called anti-inflammatory M2-like and M2 macrophages [37] (Supplementary Table S2). Similar outcomes were observed in cells exposed to the SiO_2_ nanoparticles. On the contrary, exposure to NH_2_-PS nanoparticles increased the percentage of M1 macrophages, usually considered pro-inflammatory [38]. No effects were observed upon exposure to COOH-PS. Overall, short-term exposure to the different nanoparticles, followed by a rather long recovery time in a nanoparticle-free medium, allowed us to exclude toxicity that could interfere with EV separation procedures while still allowing nanoparticle accumulation, as well as affecting macrophage polarization.

### 2.2. Extracellular Vesicle Separation and Characterization

**Figure 2:**
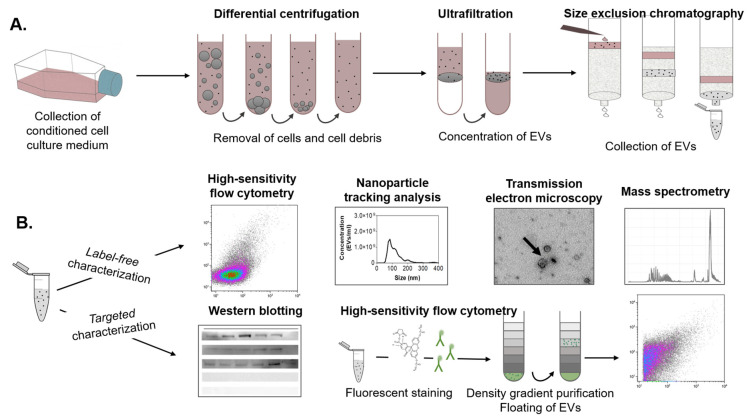
Schematic overview of the EV separation procedure and downstream analyses. (**A**) EVs were isolated from a conditioned medium of MPI cells through a combination of differential centrifugation, ultrafiltration, and size exclusion chromatography (SEC). (**B**) The eluted SEC fractions were collected for downstream analyses, including label-free high-sensitivity flow cytometry, nanoparticle tracking analysis, transmission electron microscopy, and mass spectrometry. Targeted characterization was performed by Western blotting and high-sensitivity flow cytometry. For the latter, EVs were fluorescently stained and purified by bottom-up density gradient centrifugation and subsequently measured.

After pulse and chase nanoparticle exposure of the MPI cells, the conditioned medium was collected. Then, the EVs were isolated using a combination of differential centrifugation, ultrafiltration, and, finally, size exclusion chromatography (SEC). The eluted fractions were collected for multiple downstream analyses (Figure 2), including label-free high-sensitivity flow cytometry and nanoparticle tracking analysis (NTA) to determine EV concentration and size distribution, western blot and fluorescence-based flow cytometry to characterize relevant EV markers, and transmission electron microscopy to visualize the isolated EVs and their morphology. Finally, EV protein composition was determined by liquid chromatography-mass spectrometry-based proteomics (LC-MS/MS).

**Figure 3:**
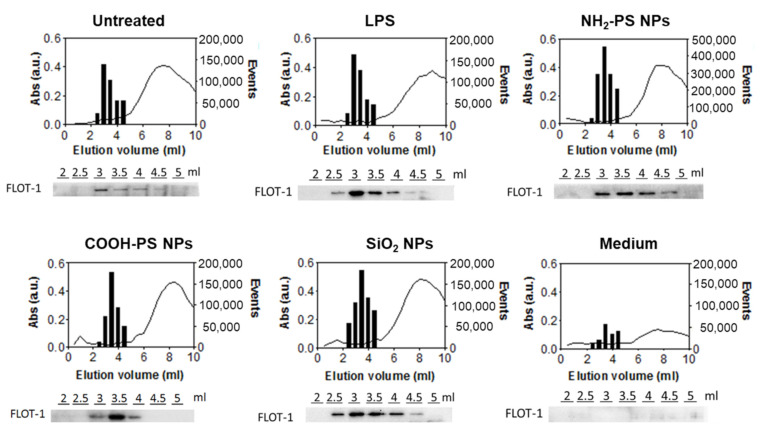
Characterization of size exclusion chromatography fractions of conditioned media from MPI cells exposed to LPS, NH_2_-PS nanoparticles, COOH-PS nanoparticles, or SiO_2_ nanoparticles. In-house-made size exclusion chromatography columns were used to process the different fractions. EVs from untreated cells (untreated) and cell culture medium without cells subjected to the same isolation procedure (medium) were used as controls. Flow cytometric analysis was performed on elution volumes between 2.5 mL and 4.5 mL to determine the number of events (bar plots). In contrast, protein absorbance at 280 nm (a.u.) was measured for all the different elution fractions between 0 and 10 mL using spectrophotometric analysis (line graph). Western blot detection of FLOT-1 was performed in individual fractions eluting between 2 and 5 mL (images below the graphs). A representative dataset is shown for every condition.

High-sensitivity flow cytometry is a frequently applied method for the detection of EVs [39,40,41]. Since EV dimensions are often smaller than the wavelength of incident laser light [42], using a 405 nm violet laser to measure side scatter (SSC) signals instead of the more common 488 m laser allowed us to strongly improve the sensitivity of label-free flow cytometry detection (Supplementary Figure S3) [43]. Thus, we used the violet SSC to characterize the relevant EV fractions collected from SEC (Supplementary Figures S3 and S4). The results showed a clear increase in the number of events compared to background events recorded from the control medium (Figure 3). Combining this with western blot analysis of flotillin-1 (FLOT-1), a lipid raft-associated membrane protein marking EVs, and absorbance measurements, used as a read-out for the elution of proteins from the cell medium, we confirmed that EVs were present in the fractions containing most scatter events [44]. More in-depth western blot analyses confirmed the presence of the EV-specific tetraspanin CD81 protein and the cytosolic ALG-2 interacting protein X (ALIX) in the EV-containing fractions (Supplementary Figure S6). In addition, the endoplasmic reticulum chaperone GRP94 and the mitochondrial protein cytochrome C were not detected, confirming the absence of cellular contamination. Transmission electron microscopy further confirmed the presence of donut-shaped EVs in the corresponding fractions (Supplementary Figure S10). Instead, in the fractions recovered from the control medium subjected to the same separation procedure, much lower protein absorption was observed. No FLOT-1 nor donut-shaped EVs were detected by western blot and TEM, respectively. Taken together, these results confirmed that the fractions with higher event counts recorded by flow cytometry contained EVs. As expected, they eluted first, followed by free proteins from the cell medium. When comparing the different nanoparticles and LPS treatment conditions, the elution profiles were similar. Nevertheless, the event rate from EVs recovered from cells exposed to the NH_2_-PS nanoparticles was higher, suggesting an increase in EV concentration.

**Figure 4:**
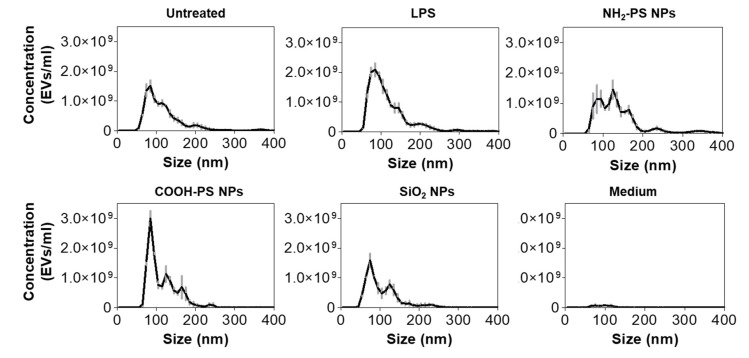
Characterization of EV size and number after exposure of MPI cells to LPS, NH_2_-PS nanoparticles, COOH-PS nanoparticles, or SiO_2_ nanoparticles. In-house-made size exclusion chromatography columns were used to isolate the EVs. EVs from untreated MPI cells (untreated) and cell culture medium without cells subjected to the same isolation procedure (medium) were used as controls. Size exclusion chromatography isolated fractions, which correspond with an elution volume between 3 mL and 4.5 mL, were pooled and analyzed using NTA. The results are shown as mean ± standard deviation of 3 replicate isolations.

In order to further compare the different cell exposure conditions, we analyzed the secreted EVs for their number, concentration, and size using NTA and combined this with a micro BCA assay to determine the total protein concentration of the EV isolates (Figure 4 and Table 1). NTA results clearly showed that exposure to LPS caused a higher yield of EV production compared to untreated cells, both in terms of concentration and protein content. However, the EVs over protein ratio was similar. Instead, in cells exposed to NH_2_-PS nanoparticles, in contrast with the results obtained by flow cytometry (Figure 3), no statistically significant increase in EV concentration was detected. However, NTA results clearly showed that the released EVs had larger sizes and higher polydispersity. The higher polydispersity can be indicative of changes in EV origin and composition. For instance, it has been shown that tumor-derived EVs have a wider range of sizes than those derived from healthy cells [45,46,47].

To further confirm the identity of the isolated EVs and determine their concentration, additional analysis was performed using high-sensitivity fluorescence-based flow cytometry (Supplementary Figures S7–S9). In this case, the EVs were double labeled with carboxyfluorescein diacetate succinimidyl ester (CFDA-SE) to stain the lumen of vesicles and with a fluorescently conjugated antibody directed to the EV membrane marker tetraspanin CD9. Bottom-up density gradient centrifugation was performed to clean the EVs from free dye [48]. In agreement with the event count obtained with label-free flow cytometry (Figure 3), the results showed that exposure of MPI cells to NH_2_-PS nanoparticles led to an increase in the concentration of CFDA-SE stained EVs in comparison to untreated cells. In addition, EV concentration was higher after exposure to LPS, but lower after exposure to SiO_2_ nanoparticles (Supplementary Figure S8). The number of EVs positive for CD9 followed a similar trend among the different treatment conditions, resulting in a percentage of CD9-positive EVs that remained relatively stable (Supplementary Figure S9).

Determining EV concentration and size distribution under different exposure conditions is technically challenging. For a correct EV enumeration, it is crucial that the smallest detectable EVs are included in the measurements. While high-sensitivity flow cytometry, both in the label-free violet scatter mode (Figure 3 and Supplementary Figures S3–S5) and after fluorescence measurements of stained EVs (Supplementary Figures S7–S9) showed an increase in EV concentration in cells exposed to NH_2_-PS nanoparticles, this increase was not observed by NTA (Figure 4). This discrepancy is probably due to the differences in the limits of detection. The minimum detection limit of EVs using scatter-based NTA is considered to be 70–90 nm [49] (depending on the scattering properties of the objects). In contrast, the minimum detection limit for high-sensitivity flow cytometry is strongly dependent on the instrument type [39]. When based on 488 nm side scattering detection, the size limit has been described to be in the range of 150 and 190 nm [49]. Additional fluorescence labeling allows the detection of smaller EVs (<100 nm), and here potentially resulted in the inclusion of the full EV diameter range [39,50,51]. Similarly, in the label-free mode, using the 405 nm laser allowed us to improve the detection sensitivity substantially. Based on these considerations, the increased and decreased EV numbers after NH_2_-PS and SiO_2_ nanoparticle exposure, respectively, observed with both label-free and fluorescence-based high-sensitivity flow cytometry, can be assumed to be genuine.

Overall, the detailed characterization performed showed that the EVs released by the macrophages following exposure to nanoparticles were different in terms of size, polydispersity, and concentration than those released by untreated cells or following stimulation by LPS, as well as depending on the nanoparticle type.

### 2.3. Protein Composition of EVs

**Figure 5:**
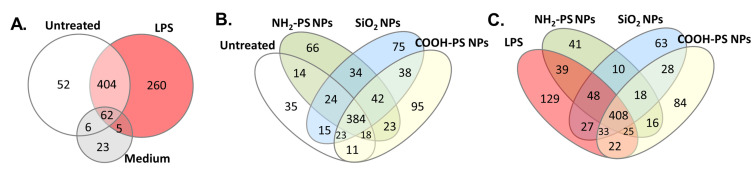
Protein analysis of EVs from MPI cells after exposure to LPS, NH_2_-PS nanoparticles (NPs), COOH-PS nanoparticles, or SiO_2_ nanoparticles measured by LC-MS/MS. Commercial size exclusion chromatography columns were used to isolate the EVs. (**A**–**C**) EVs from untreated MPI cells (untreated) and cell culture medium without cells subjected to the same isolation procedure (medium) were used as controls. The Venn diagrams show the overlap between all proteins detected under the different conditions in at least one of the samples obtained from 4 replicate isolations. The full results and list of proteins for each replicate sample for all conditions are included in Supplementary Table S3.

To further characterize EV composition under the different MPI cell exposure conditions, we used liquid chromatography-tandem mass spectrometry (LC-MS/MS) to identify the proteins in the EVs. The results show that among the identified proteins, EV-related proteins such as actin, synthenin-1, and annexin types were highly abundant (see Supplementary Table S3 for the identified proteins). LC-MS/MS measurements of SEC fractions of the cell culture medium containing 2.5% EV-depleted FBS (medium control) resulted predominantly in the identification of background proteins, such as keratin types. Some EV-related proteins were also identified. However, it is known that commercially available EV-depleted FBS may still contain residual bovine EVs [52]. Importantly, a comparison of the identified proteins demonstrates that there was a substantial difference between the EVs produced by untreated cells and in the medium background (Figure 5A).

Moreover, when comparing the composition of EVs produced by untreated cells and cells exposed to LPS or the different nanoparticles, other interesting differences were observed (Figure 5B,C). To allow assessment of statistically significant protein enrichment (*p* ≤ 0.05) in MPI cell-derived EVs following nanoparticle or LPS treatment compared to untreated cells, protein quantification was performed using an in-house developed algorithm [53].

**Figure 6:**
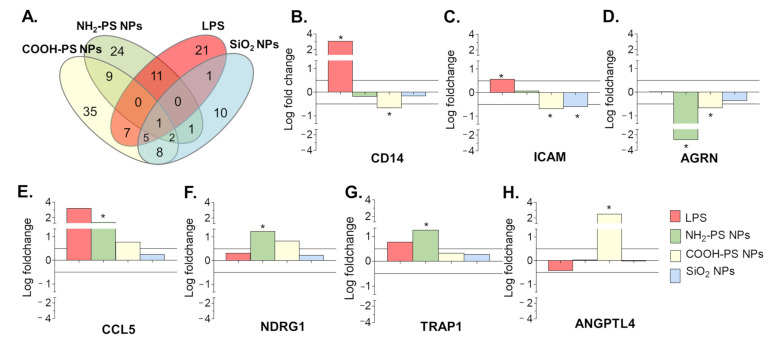
Proteins enrichment in EVs produced by MPI cells in response to exposure to LPS, NH_2_-PS nanoparticles (NPs), COOH-PS nanoparticles, or SiO_2_ nanoparticles, as compared to the untreated cells. Commercial size exclusion chromatography columns were used to isolate the EVs. (**A**) Venn diagram presenting the statistically significant enriched proteins in the EVs produced by MPI cells in response to exposure to LPS or the different nanoparticles, compared to the EVs from untreated control cells (with *p*-values lower or equal to 0.05). (**B**–**H**) Selection of most statistically significant differences in protein abundance, calculated from four replicate isolations per condition (see Methods for details). Differences in composition were considered biologically significant when *p*-values were lower or equal to 0.05 (indicated with *) and the logarithmic fold changes were higher than |0.50|. The complete results of the statistical analysis performed on all common proteins identified in the EVs from untreated control cells and cells exposed to LPS or the different nanoparticles in 4 independent isolations are included in Supplementary Table S4.

The results show that COOH-PS nanoparticles induced a most distinct EV profile (67 differentially expressed proteins), followed by NH_2_-PS (48), LPS (46), and SiO_2_ nanoparticles (28). Besides overlapping proteins among the different conditions, each also showed unique signatures (Venn diagram in Figure 6A and Supplementary Table S4 for the full results). A selection of the most significant variations in EV composition is given in Figure 6B–H. Exposure to LPS resulted in EVs containing significantly more CD14 and ICAM-1, among other proteins. This agrees with other studies, which linked the presence of CD14 and ICAM-1 on EVs with an inflammatory cell response [54,55,56]. Exposure to NH_2_-PS and COOH-PS nanoparticles both resulted in the release of EVs with a significant decrease in Agrin (AGRN), a basement membrane glycoprotein that was previously shown to be absent in leukemia-derived EVs [57]. Similarly, exposure to COOH-PS and SiO_2_ nanoparticles resulted in a loss of ICAM-1 on the EVs. Agrin and ICAM-1 are involved in the uptake of EVs by their recipient cells, suggesting that nanoparticle exposure might interfere with the uptake of EVs released by the macrophages [54,55,57,58]. Furthermore, the EVs released following exposure to NH_2_-PS nanoparticles showed a statistically significant increase in the C-C motif chemokine ligand 5 (CCL5), TNF Receptor Associated Protein 1 (TRAP1) and *n*-myc downstream regulated 1 protein (NDRG1), suggesting a more inflammatory EV phenotype [54,59]. This is possibly related to the mechanisms of toxicity activated by these nanoparticles at higher concentrations, where cell death was observed after mitochondrial hyperpolarization, endoplasmic reticulum stress, and increased ROS production, however, without classic apoptotic features [34]. COOH-PS nanoparticles, on the other hand, caused an increase in the angiopoietin-like protein 4 (ANGPTL4) content of the EVs, which, e.g., was also shown to be upregulated in response to starvation conditions [60]. It is interesting to notice that several changes were also observed in response to COOH-PS, for which no evident toxicity was observed, even after exposure to higher nanoparticle concentrations. This further confirms that changes in the released EVs and their composition may allow a better understanding of subtle responses induced by nanoparticles, including for those many cases where nanoparticles seem generally well tolerated. Similarly, it will be interesting to determine the functional relevance of the observed alterations and potential implications, for instance, in relation to the long-term side effects of these nanoparticles.

Overall, the proteomic analysis clearly showed that exposure to sub-toxic concentrations of the selected nanoparticles induced the release of EVs of altered composition depending on the nanoparticle type, which is known to have a different impact on cells. Other parameters and nanoparticle properties are likely to affect in similar ways the EVs released by macrophages. For instance, exposure to nanoparticles of different sizes and shapes is likely to lead to changes in the released EVs. For nanoparticles subjected to degradation and/or ion release, additional effects may be distinguished due to the degradation products and the released ions. At a broader level, the observed differences in released EVs suggest that exposure to nanoparticles may interfere with intercellular communication mechanisms as well. Analyzing similar differences in EV characteristics and composition can provide novel information on subtle responses to nanoparticles also for cases where no evident toxicity was reported, and more specifically on nanoparticle impact on intercellular communication. Functional studies should be further examined to fully understand the effect of changes in the EV release profile.

## 3. Materials and Methods

### 3.1. Cell Culture

Max Planck Institute (MPI) cells were kindly provided by Prof. Dr. Gyorgy Fejer (Plymouth University, UK) [29]. For routine subculturing floating cells in the supernatant were collected and combined with the adherent cells detached with 1.5 mM EDTA (Merck Millipore, Darmstadt, Germany) in phosphate buffer saline (PBS, Gibco, Grant Island, NY, USA). Combined cells were centrifuged and resuspended in a complete cell culture medium (CCM) composed of RMPI 1640 (Gibco) supplemented with 10% heat-inactivated fetal bovine serum (FBS, Gibco) and 20 ng/mL GM-CSF (Peprotech, Bio-Connect, Huissen, The Netherlands). Cells were seeded at a density of 20,000 cells/cm^2^ and subcultured weekly. The cell culture medium was refreshed after 5 days. All experiments were performed with cells between passage 6 and passage 12.

### 3.2. Nanoparticle Characterization and Exposure to Cells

The nanoparticles used for this work were 50 nm plain silica (SiO_2_, Kisker Biotech, Steinfurt, Germany), 50 nm carboxylated polystyrene (COOH-PS, Polysciences Europe GmbH, Hirschberg an der Bergstrasse, Germany), and 50 nm amino-modified polystyrene (NH_2_-PS, Bang Laboratories, Fishers, IN, USA) (all unlabeled). To confirm nanoparticle uptake by cells and optimize nanoparticle exposure time, green fluorescently labeled 50 nm plain silica nanoparticles (Sicastar^®^ green, MicroMod, Wallingford, CT, USA) were used. Nanoparticle dispersions were prepared by diluting the concentrated stock dispersions in complete CCM supplemented with 20 ng/mL GM-CSF immediately prior to exposure of the cells. Cells were exposed to nanoparticles by replacing the old medium with freshly prepared nanoparticle dispersions. Dispersions of 100 µg/mL nanoparticles in water, PBS, and CCM were characterized by means of dynamic light scattering and zeta potential determination using a Malvern Zeta Sizer ZS (ZEN 3600, Malvern Instruments, Malvern, UK). Additionally, the dispersions in CCM were characterized after incubation for 24 h at 37 °C and 5% CO_2_ to confirm stability in CCM in the conditions used for experiments with cells. For each sample, ten runs of three measurements of 10 s at 25 °C were performed. The results are the mean ± standard deviation.

To assess the effects of nanoparticle exposure on the cellular metabolic activity of the MPI cells and determine a sub-toxic exposure regimen, an MTT assay was performed. MPI cells were seeded (40,000 cells per well) in a 96-well plate, and 48 h after seeding were exposed for the indicated times to 100 ng/mL lipopolysaccharide from *E. coli* serotype 055:B5 (LPS, Sigma-Aldrich, St. Louis, MO, USA) or nanoparticles (25–50–100 µg/mL) as described before (100 µL dispersions). After exposure, the medium containing the nanoparticles was removed, and 100 µL of MTT (3–(4,5-dimethylthiazol-2-yl)–2,5-diphenyltetrazolium bromide) reagent (Sigma-Aldrich, St. Louis, MO, USA) dissolved to a final concentration of 500 µg/mL in EV-depleted cell culture medium was applied to the cells for 30 min at 37 °C and 5% CO_2_. Thereafter, the medium containing the MTT reagent was removed, and the precipitated formazan crystals were solubilized using 100 µL dimethyl sulfoxide (DMSO). The absorbance was read out at 550 nm (ThermoMax, Molecular Devices, Sunnyvale, CA, USA). The results are shown as the mean ± standard deviation of 3 replicate samples per condition, normalized by the results obtained in untreated control cells.

### 3.3. Confocal Fluorescence Microscopy

To visualize the uptake of fluorescent SiO_2_ nanoparticles in MPI cells, 250,000 cells were seeded on 60 mm glass bottom dishes (MatTek, Bratislava, Slovak Republic). After 48 h, cells were exposed for 2 h to the nanoparticles by replacing the medium with the freshly prepared SiO_2_ nanoparticle dispersions in CCM (25 µg/mL). Cells were washed with fresh CCM two times and once with PBS. Image acquisition was performed immediately using an SP8 Leica confocal fluorescence microscope (Leica Biosystems, Deer Park, IL, USA) and the 488 nm laser line for visualization of the fluorescent nanoparticles.

### 3.4. Annexin V–Cell Permeability Assay and Phenotyping of MPI Cells

In order to assess the effect of nanoparticle exposure on the phenotype of MPI cells, 200,000 cells were seeded on 12-well plates. After 48 h, cells were exposed to a volume of 2 mL containing 25 µg/mL nanoparticles in CCM, 100 ng/mL LPS in CCM, or CCM alone for 1 h. Thereafter, cells were washed with 2 mL of CCM and 2 mL PBS, and the medium was replaced by 2 mL of RMPI 1640 supplemented with 2.5% EV-depleted FBS (Gibco) and 20 ng/mL GM-CSF. After 23 h, MPI cells were harvested using 1.5 mM EDTA in PBS. The exposure of phosphatidylserine in apoptotic cells and dead cells was detected using a Pacific Blue Annexin V/SYTOX AADvanced Apoptosis Kit (Thermo Fisher Scientific, Waltham, MA, USA). Briefly, the recovered cells were stained according to the manufacturer’s guidelines. The cell fluorescence intensity was measured immediately using a CytoFLEX S flow cytometer: a 405 nm laser was used to excite Annexin V, and a 488 nm laser for SYTOX AADvanced.

For cell phenotyping, detached MPI cells (see above) were washed with 5 mM EDTA + 1% FBS in PBS and collected by centrifugation for 5 min at 500 rcf. Thereafter, cells were stained with 0.2 µg/mL anti-mouse CD206-Alexa Fluor 647 (BioLegend, San Diego, CA, USA, # 141711) and 0.2 µg/mL anti-mouse MHCII-APC/Cy7 (Biolegend, # 307617) diluted in with 5 mM EDTA + 1% FBS in PBS for 30 min at 4 °C. Thereafter, cells were washed two times with 5 mM EDTA + 1% FBS in PBS and once with PBS. The cell fluorescence intensity was measured immediately using a CytoFLEX S flow cytometer (Beckman Coulter, Brea, CA, USA) and a 638 nm laser was used to excite Alexa^®^ Fluor 647 and APC/Cy7. The results are presented as the mean percentage of positive cells ± standard deviation of 2 replicate experiments.

### 3.5. Extracellular Vesicle Separation

MPI cells were seeded at a density of 7 × 10^6^ cells in a T75 cm^2^ cell culture flask (Greiner Bio-One, Frickenhausen, Germany) in CCM. After 3 days, when reaching 80% confluency, cells were exposed to a volume of 15 mL containing 25 µg/mL nanoparticles in CCM, 100 ng/mL LPS in CCM, or CCM alone for 1 h. Thereafter, cells were washed with 15 mL of CCM and 15 mL PBS, and the medium was replaced by 15 mL of RMPI 1640 supplemented with 2.5% EV-depleted FBS (Gibco) and 20 ng/mL GM-CSF. After 23 h, the cell medium was collected. The cell medium was purified in order to remove cells and cellular debris by means of differential centrifugation steps at 300 rcf, 400 rcf, 500 rcf, and 1000 rcf for 10 min at 4 °C in a swinging bucket Rotanta 460 R centrifuge (Hettich Benelux B.V., Geldermalsen, The Netherlands) using 15 mL polypropylene tubes (Greiner Bio-One, Kremsmuster, Austria, #188271) with maximal brake. Next, the medium was centrifuged at 3750 rcf for 30 min at 4 °C in a swinging bucket Rotanta 460 R centrifuge using 15 mL polypropylene tubes with maximal brake. The purified cell medium was concentrated using ultrafiltration. Per separation procedure, 15 mL of medium was concentrated to approximately 300 µL using regenerated cellulose membrane 100 kDa MWCO Amicon Ultra-15 Centrifugal Filter Units (Merck Millipore, Burlington, MA, USA, #UCF910024) in a swinging bucket Rotanta 460 R centrifuge at 3750 rcf for 30 min at 4 °C with maximal brake. EVs were isolated from the concentrated cell medium using size exclusion chromatography (SEC). Two different approaches were used: in-house made size exclusion chromatography columns (Bio-Rad Laboratories B.V., Veenendaal, The Netherlands, #7321010) containing a bed volume of 10 mL of Sepharose CL-4B (Sigma-Aldrich, #CL4B200) or commercial qEV column (Izon) with a 70 nm pore size and a bed volume of 10 mL. Fractions of 500 µL were collected in protein LoBind Eppendorf tubes (Eppendorf, Hamburg, Germany, #022431081), and collection started immediately after placing the sample on the column. Degassed and 0.22 µm filtered PBS without Ca^2+^ and Mg^2+^ (Gibco, #21600–069) was used as the elution buffer. Protein concentrations of the isolated EV fractions were determined by the Micro BCA™ Protein Assay Reagent Kit (ThermoFisher Scientific) at 37 °C according to the manufacturer’s guidelines. The absorbance was read out at 561 nm (Clariostar, BMG Labtech, Ortenberg, Germany). A standard curve of serially diluted Bovine Serum Albumin (ThermoFisher Scientific) in 0.22 µm filtered PBS was used. In addition, absorbance spectrophotometry of the isolated fractions was performed using a NanoDrop™ One Instrument (ThermoFisher Scientific) measuring at 280 nm.

### 3.6. Nanoparticle Tracking Analysis

EV size and concentrations were determined by means of scatter-based nanoparticle tracking analysis (NTA) using a NanoSight LM14 instrument (NanoSight, Malvern Panalytical, Worcestershire, UK) and NTA software version 3.0 according to the manufacturers’ guidelines. After purification of EVs, performed as described above, fractions 6, 7, and 8 from the gravity column (roughly corresponding to elution volumes 3–4.5 mL) were pooled and diluted 1 in 10 in 0.22 µm filtered PBS prior to injection into the sample chamber. For every sample, three videos of 60 s were recorded (1499 frames with 25 frames/s), and a camera level of 14 was used. For the analysis, a detection threshold of 4 was used. The number of particles per frame ranged from 40 to 90 for the EV samples and ~3 for the medium control. The size distributions are shown in Figure 4. The sizes (modes) and concentrations shown in Table 1 are the mean ± standard deviation from 3 replicate EV isolations per condition.

### 3.7. Transmission Electron Microscopy 

EV morphology was examined by transmission electron microscopy (TEM) imaging. The sample preparation was based on previous research [61]. In brief, three droplets of the EV fractions corresponding to an elution volume between 3 and 4 mL were placed on a clean Parafilm. Afterward, a carbon-coated TEM grid was placed on top of the droplets and allowed to stand for 60 min to adsorb the fluid. The grid with adherent EVs was washed three times with 0.22 µm filtered PBS for 2 min and five times with 0.22 µm filtered ultrapure water for 2 min. Next, EVs were fixed with 2% glutaraldehyde for 10 min and then washed five times with filtered water for 2 min. Grids were transferred to 2% uranyl acetate and allowed to stand for 15 min. Thereafter, grids were incubated in 0.13% methylcellulose (K5–8) and 0.4% uranyl acetate for 10 min. Finally, grids were dried at room temperature before examination with Tecnai G2 Spirit BioTWIN (FEI, Hillsboro, OR, USA).

### 3.8. Western Blotting

Equal volumes of each fraction and 10 µg of MPI cell lysate were heated at 95 °C in Laemmli sample buffer containing a final concentration of 5% sodium dodecyl sulfate (SDS), 15 mM Tris, 0.025% glycerol, and 1.25% β-mercaptoethanol (all Sigma-Aldrich). Samples were subjected to gel electrophoresis on a 12% SDS-polyacrylamide gel for 1 h at 100 V. Subsequently, the proteins were transferred to a polyvinylidene difluoride (PVDF) Western Blotting Membrane (Roche Diagnostics, Almere, The Netherlands), which was pre-wetted in methanol for 60 s, for 1.5 h at 100 V using a transfer buffer containing 25 mM Tris, 192 mM glycine and 20% (*v*/*v*) methanol (all Sigma-Aldrich). Thereafter, membranes were blocked in 5% BSA (Sigma-Aldrich) for flotillin-1 and GRP94 or 5% non-fat dry milk (Bio-Rad) for CD81, ALIX, and cytochrome C in Tris-buffer saline (TBS) containing 0,1% Tween20 (Sigma-Aldrich) (TBS-T) for 2 h with gentle rolling at room temperature. Thereafter, the primary anti-mouse antibodies against flotillin-1 (Cell Signaling Technology, Danvers, MA, USA, #18634, dilution 1:500), CD81 (Cell Signaling Technology, #10037, dilution 1:500), ALIX (Cell Signaling Technology, #2171, dilution 1:1000), GRP94 (Cell Signaling Technology, #2029, dilution 1:1000) and cytochrome C (Santa Cruz Biotechnology, Dallas, TX, USA, #sc-13156, dilution 1:1000) were added overnight in their respective blocking buffer. After washing three times for 5 min with TBS-T, an HRP-conjugated goat anti-rabbit IgG (Cell Signaling, #7074, dilution 1:1000) or an HRP-conjugated donkey anti-mouse IgG (Jackson Immunoresearch, West Grove, PA, #715-035-151, dilution 1:5000) was added for 2 h at room temperature in their respective blocking buffer. After washing three times for 5 min with TBS-T and three times for 5 min with TBS, bands were detected using VisiGlo Prime Chemiluminescent Substrate Kit (VWR, Solon, OH, USA).

### 3.9. Flow Cytometry of EVs

Flow cytometry analysis based on scatter was performed using a CytoFLEX S flow cytometer (Beckman Coulter). The flow cytometer was equipped with 405 nm, 488 nm, 561 nm, and 638 nm lasers. The device utilizes a sensitive side scatter (violet 405 nm side scatter) as a trigger parameter, which results in a higher particle resolution allowing for small particle detection down to at least 150 nm, as described by Wisgrill and coworkers [62]. The CytoFLEX was operated according to the manufacturers’ recommendations. Briefly, in order to set the measurements for vesicle detection, the threshold of the trigger signal of the violet side scatter (Violet SSC) was set to 2000. The gain of the Violet SSC was adjusted to 15. The sample line was cleaned with fresh distilled water for 10 min prior to the measurements and for 2 min between samples at a flow rate of 60 µL/min. Samples were measured at a flow rate of 10 µL/min. Fluorescent Megamix-Plus SSC and Megamix-Plus FSC beads (BioCytex, Marseille, France, #7803 and #7802) were used for standardization of small particle detection.

In addition, fluorescently labeled EVs were analyzed using a BD Influx flow cytometer equipped with a high-power 488 nm laser (200 mW) and a small-particle detector for high-sensitivity forward scatter detection. The device utilizes a highly sensitive FL-1 fluorescence trigger (bandpass filter BP530/40) to measure the EVs. For this, 200 µL of EV fractions were stained using 10 µM Vybrant carboxyfluorescein diacetate, succinimidyl ester (CFDA-SE) Cell Tracer (ThermoFisher Scientific, #V12883) for 1 min in the dark at room temperature. Thereafter, to allow for EV identification, samples were incubated with 0.5 µg anti-mouse CD9-BB700 antibody (BD Biosciences #742131) for 1 h in the dark at room temperature. Prior to incubation with EV samples, the antibody dilution was centrifuged for 5 min at 16,000 rcf at 4 °C to remove potential antibody aggregates using a Sigma 3–30KS centrifuge (VWR). Stained EVs were purified by bottom-up density gradient centrifugation using an iodixanol gradient (OptiPrep, StemCELL Technologies, Vancouver, Canada, #07820). Briefly, samples with a final volume of 300 µL were mixed with 1 mL of 60% iodixanol. This mixture was overlaid with 700 µL of 40% iodixanol, 700 µL of 30% iodixanol, and 2 mL of 10% iodixanol. Iodixanol dilutions were prepared by dilution of the 60% iodixanol to 40% iodixanol using a homogenization buffer containing 6 mM EDTA, 60 mM Tris-HCl, and 0.25 mM sucrose (pH 7.4), and subsequent dilutions were performed by dilution of 40% iodixanol to 30% iodixanol and 10% iodixanol using a homogenization buffer containing 1 mM EDTA, 10 mM Tris-HCl and 0.25 mM sucrose (pH 7.4). Samples were centrifuged for 14 h at 367,600 rcf at 4 °C in 5 mL open-top thin-wall polyallomer tubes (Beckman Coulter, Brea, CA, USA, #103242) without brake using an Optima XPN-80 ultracentrifuge (Beckman Coulter, *k*-factor: 48.5). Fractions of 470 µL were collected and measured at a flow rate of ±10 µL/min. The threshold of the FL-1 fluorescent channel (BP530/40) to detect CFDA-SE stained EVs was set at 0.30 arbitrary units. Yellow-Green fluorescent beads of 100 and 200 nm (ThermoFisher Scientific) were used for standardization of small particle detection (Supplementary Figure S7A,B) and also measured with BP530/40. Fluorescent signals of CD9-positive EVs were detected with BP692/40. In order to confirm that individual EV analyses by high-sensitivity flow cytometry were reliable for concentration measurements, the absence of swarming was validated at the operational range by measuring samples serially diluted in PBS. The event rate and the concentration of EVs were observed to increase proportionally (Supplementary Figure S7C). Finally, the vesicular nature of recorded CFDA-SE stained EVs was confirmed by a high decrease in event number after adding the detergent Triton-X100 (0.1% *v*/*v*) for 15 min at room temperature (Supplementary Figure S7D).

### 3.10. Mass Spectrometry

Mass spectrometry was used to identify the proteins in samples prepared from 4 replicate EV isolations from independent nanoparticle exposure experiments. Size exclusion chromatography fractions corresponding to an elution volume between 3 and 4 mL were pooled and vacuum dried in 2 mL protein LoBind Eppendorf tubes. A tert-butyl methyl ether (MTBE) lipid extraction method was applied to the EV pellets as described before [63]. EV pellets were resuspended in 260 µL methanol and vortexed for 10 sec. Thereafter, 1 mL of MTBE (Sigma-Aldrich) was added, and samples were mixed for 1 h at room temperature. Phase separation was performed by adding 260 µL water, and samples were incubated for 10 min at room temperature. Next, samples were centrifuged at 1000 rcf for 10 min resulting in two phases: a lower hydrophilic phase and an upper lipophilic phase. The lipophilic phase (1 mL) was removed, and the lower hydrophilic and protein layer were vacuum dried. Next, samples were resuspended in 75 µL of 5M urea, vortexed, and sonicated for 10 min. This step was repeated. Proteins were reduced at 60 °C for 30 min in a final concentration of 5 mM dithiothreitol. Proteins were subsequently alkylated at room temperature for 30 min in a final concentration of 20 mM iodoacetamide. Next, 680 µL of 100 mM ammonium bicarbonate was added. One µg trypsin was added per 40 µg of proteins, and trypsin digestion was carried out overnight at 37 °C. Digests were desalted using Pierce C18 spin columns (ThermoFisher Scientific) according to the manufacturer’s guidelines.

For LC-MS/MS, the eluted peptides were vacuum dried and dissolved in mobile phase A, containing 2% acetonitrile and 0.1% formic acid to a final concentration of 0.1 µg/µL, and spiked with 0.5 fmol Glu-1-fibrinopeptide B (Glu-fib, Protea biosciences, Morgantown, WV, USA). A total of 1 µg of protein was loaded on the column, and the peptide mixture was separated by reversed-phase chromatography on a nanoACQUITY system (Waters) using a nanoACQUITY UPLC Symmetry C18 Trap Column (100Å, 5 µm, 180 µm × 20 mm, 2G, V/M, 1/pkg) (Waters, Milford, Massachusetts, USA) connected to an ACQUITY UPLC PST C18 nanoACQUITY Column (10K psi, 130Å, 1.7 µm, 100 µm × 100 mm, 1/pkg) (Waters). A linear gradient of mobile phase B (0.1% formic acid in 98% acetonitrile) from 1 to 45% in 80 min was followed by a steep increase to 100% mobile phase B in 5 min. A steep decrease to 1% mobile phase B was achieved in 5 min, and 1% mobile phase B was maintained for 35 min. The flow rate was 750 nl per minute. Liquid chromatography was followed by tandem MS (LC-MS/MS) performed on a Q-Exactive plus mass spectrometer (ThermoFisher Scientific) using a nanospray ion source (ThermoFisher Scientific). Full scan spectrum (350 to 1850 m/z, resolution 70,000, automatic gain control 3E6, maximum injection time 100 ms) was followed by high-energy collision-induced dissociation (HCD) tandem mass spectra with a run time of 120 min. Peptide ions were selected for fragmentation by tandem MS as the 20 most intense peaks of a full-scan mass spectrum. HCD scans were acquired in the Orbitrap (resolution 17,500, automatic gain control 1E5, maximum injection time 80 ms). Proteome Discoverer (2.1) software (ThermoFisher Scientific) was used to perform database searching against the Uniprot Mouse database (mus_musculus_reviewed_20180203.fasta) using both Sequest and Mascot algorithms (in-house server). Searches were performed with the following settings: precursor mass tolerance of 5 ppm and fragment mass tolerance of 0.02 Da. Digestion by trypsin and two missed cleavage sites are allowed. Carbamidomethyl modifications were defined as fixed modifications and phosphorylations (Ser, Thr, Tyr) and oxidations (of methionine) as dynamic modifications. The results were filtered with the following parameters: only high confident peptides with a global false-discovery rate (FDR) <1% based on a target-decoy approach and first-ranked peptides were included in the results.

An analytical workflow to extract time-intensity chromatograms from high-resolution MS1 data was used in order to quantify all highly confident identified peptides. Thereafter, data were normalized by classical quantile normalization, starting from the peak intensities extracted from the MS1 data.

### 3.11. Statistical Analyses

Statistical analyses in Table 1 were performed by GraphPad Prism version 5.00 (GraphPad Prism version 5.00 for Windows, San Diego, CA, USA). Mean values and standard deviation (SD) were calculated. One-way analysis of variance (ANOVA) was performed, and results were considered significantly altered compared to the untreated control when *p* < 0.05 using Tukey’s Multiple Comparison Test.

Statistical analyses of the proteomics data were performed on the protein level, with only unique peptide sequences considered. A fold change per protein was determined by calculating for each peptide the average peak intensity over four replicate isolations and using this to obtain the fold change in peptide abundance between untreated control cells and cells exposed to LPS or the different nanoparticles. Next, for each protein, the median of all fold changes of all peptides was determined. Differences in composition were tested using ANOVA and considered biologically significant when *p*-values were lower or equal to 0.05, and the logarithmic fold changes were higher than |0.50|. *p*-values were corrected for multiple testing via false discovery rate multiple testing.

### 3.12. EV-TRACK

All relevant data from the experiments were added to the EV-TRACK knowledgebase (EV-TRACK ID: EV180067) [64].

## Figures and Tables

**Table 1:** Effect of MPI cell treatment with nanoparticles on EV size and concentration and total protein content. Fractions recovered from size exclusion chromatography with an elution volume between 2.5 mL and 4.5 mL were pooled and analyzed using scatter-based NTA to assess the mode of EV hydrodynamic diameter (nm) after exposure of MPI cells to LPS or nanoparticles together with their concentration (EVs/mL). EVs from untreated MPI cells (untreated) and cell culture medium without cells subjected to the same isolation procedure (medium) were used as controls. Total protein concentration (µg/mL) was determined in the same fractions using the microBCA method. All values are reported as the mean ± SD from 3 replicate EV isolations per condition for the NTA measurements and mean ± SD from 5 replicate EV isolations per condition for the protein content. Statistically significant values compared to EVs from untreated cells are marked with * for *p* < 0.05, ** *p* < 0.01, and *** *p* < 0.001.

Condition	Diameter (nm)	Concentration (EVs/mL)	Protein Concentration (µg/mL)	Ratio (EVs/µg Protein)
Untreated	116 ± 3	(103 ± 5) × 10^7^	4.9 ± 0.5	~21.0 × 10^7^
LPS	114 ± 2	(157 ± 8) × 10^7^ ***	7.4 ± 1.1 **	~21.2 × 10^7^
NH_2_-PS nanoparticles	139 ± 9 **	(122 ± 2) × 10^7^	5.6 ± 1.0	~21.8 × 10^7^
COOH-PS nanoparticles	112 ± 1	(129 ± 2) × 10^7^ *	5.6 ± 0.4	~23.3 × 10^7^
SiO_2_ nanoparticles	102 ± 6	(87 ± 3) × 10^7^	5.7 ± 2	~15.3 × 10^7^
Medium	98 ± 6	(5 ± 1) × 10^7^ ***	1.1 ± 0.2 ***	~4.5 × 10^7^

## Data Availability

The data presented in this study are available on request from the corresponding author. All relevant data from the experiments were added to the EV-TRACK knowledgebase (EV-TRACK ID: EV180067) [64].

## Supplementary Materials

The following supporting information can be downloaded at: https://www.mdpi.com/article/10.3390/ijms24010260/s1. Supplementary Material file with additional nanoparticle characterization; metabolic activity apoptotic response and polarization of MPI cells after nanoparticle exposure; additional high-sensitivity flow cytometry plots; Western blot of EVs produced by MPI cells after exposure to nanoparticles; TEM images of the EVs; Supplementary excel files with the complete list of proteins identified in the EVs and their fold change in the EVs released by treated MPI cells in comparison to those released by untreated MPI cells. References [34,38,65,66] are cited in the supplementary materials.
