# Supplementary material for: Macrophages Release Extracellular Vesicles of Different Properties and Composition Following Exposure to Nanoparticles
Source: Int J Mol Sci. 2022 Dec 23;24(1):260. doi: 10.3390/ijms24010260 (PMC9820242; doi:10.3390/ijms24010260)
Supplement: Supplementary file 1 [file ijms-24-00260-s001.zip › Deville et al SI.pdf]

Supporting Information

Macrophages release extracellular vesicles of different properties and composition following exposure to nanoparticles

Sarah Deville [1,2,3,†], Hector Garcia Romeu [1], Eline Oeyen [2,4], Inge Mertens [2,4], Inge Nelissen [2,*] and Anna Salvati [1,*]

[1] Department of Nanomedicine & Drug Targeting, Groningen Research Institute of Pharmacy, University of Groningen, A. Deusinglaan 1, 9713AV Groningen, The Netherlands
[2] Health Unit, Flemish Institute for Technological Research, Boeretang 200, 2400 Mol, Belgium
[3] Biomedical Research Institute, Hasselt University, Agoralaan building C, 3590 Diepenbeek, Belgium
[4] Centre for Proteomics (CfP), University of Antwerp, Groenenborgerlaan 171, 2020 Antwerp, Belgium

Supplementary Tables & Figures

Supplementary Table 1: Nanoparticle characterisation. Size distribution by dynamic light scattering and zeta potential measurements of 50 nm NH_2-PS, COOH-PS and SiO_2 nanoparticles, dispersed at 100 µg/ml in water, PBS and complete cell culture medium with serum (CCM). The dispersions were characterised immediately after preparation and after 24 hours of incubation at 37°C and 5% CO_2. All values are reported as the mean ± SEM of three independent replicate measurements. Data reproduced as a reference from *Deville et al* [1]. The results confirmed that stable dispersions were obtained in the conditions used for experiments with cells.

Nanoparticles	Medium	Diameter (nm)	PDI†	Zeta Potential (mV)
NH_2-PS	H_2O	56 ± 1*	0.08 ± 0.02	35 ± 1
	PBS	103 ± 7*	0.36 ± 0.01	18 ± 1
	CCM fresh	161 ± 31‡	-	-8 ± 1
	CCM 24 hours	141 ± 25‡	-	-9 ± 1
COOH-PS	H_2O	60 ± 1*	0.19 ± 0.02	-52 ± 1
	PBS	54 ± 1*	0.04 ± 0.02	-29 ± 2
	CCM fresh	112 ± 2‡	-	-8 ± 1
	CCM 24 hours	118 ± 6‡	-	-7 ± 1
SiO_2	H_2O	68 ± 1*	0.20 ± 0.01	-17 ± 3
	PBS	76 ± 1*	0.27 ± 0.03	-17 ± 3
	CCM fresh	101 ± 9‡	-	-9 ± 1
	CCM 24 hours	155 ± 7‡	-	-9 ± 1

* z-average hydrodynamic diameter extracted by cumulant analysis of the data.

† Polydispersity index (PDI) from cumulant fitting of the data.

‡ Average hydrodynamic diameter determined from CONTIN size distribution.

Supplementary Table 2: Polarization of MPI cells upon nanoparticle treatment. Flow cytometry analysis of CD206 and MHCII of MPI cells after exposure for 1 hour to fresh medium (untreated cells), 100 ng/ml LPS, or 25 µg/ml nanoparticles (NH_2 PS, COOH PS or SiO_2) and 23 hours of recovery in EV-depleted medium. M1 macrophages are defined as CD206(lo) MHCII(hi), M2 macrophages as CD206(hi) MHCII(hi) and anti-inflammatory macrophages (also known as M2-like) as CD206(hi) MHCII(lo)[2]. Changes in CD206 and MHCII illustrate the dynamics of macrophage polarization upon treatment condition. The results are presented as the percentage of positive cells ± standard deviation of two replicate experiments. The results show that exposure to LPS decreased the number of the M2-like and M2 macrophages. Similar outcomes were observed in cells exposed to the SiO_2 nanoparticles. On the contrary, exposure to NH_2-PS nanoparticles increased the percentage of M1 macrophages. Exposure to COOH-PS nanoparticles did not affect macrophage polarization.

	**CD206(hi) MHCII(lo) M2-like**	**CD206(hi) MHCII(hi) M2**	**CD206(lo) MHCII(lo)**	**CD206(lo) MHCII(hi) M1**
Untreated cells	79 ± 3 %	10 ± 1 %	8 ± 3 %	3 ± 1 %
LPS	63 ± 14 %	3 ± 2 %	29 ± 15 %	4 ± 1 %
NH_2 PS nanoparticles	82 ± 1 %	14 ± 2 %	3 ± 2 %	7 ± 1 %
COOH PS nanoparticles	81 ± 1 %	9 ± 1 %	7 ± 1 %	3 ± 0 %
SiO_2 nanoparticles	75 ± 7 %	6 ± 1 %	15 ± 7 %	3 ± 1 %

Supplementary Table 3: Proteins identified by LC-MS/MS measurement in EVs released by untreated MPI cells and MPI exposed to 100 ng/ml LPS, or 25 µg/ml NH_2-PS, COOH-PS, or SiO_2 nanoparticles. Commercial size exclusion chromatography columns were used to isolate the EVs. All the proteins identified in the samples obtained from 4 replicate isolations are included. The results from a sample of medium subjected to the same isolation procedures are also included (medium). (Separate excel file)

Supplementary Table 4: Overview of fold changes and p-values of the statistical analysis performed on all proteins identified in the EV isolated from MPI cells exposed to 100 ng/ml LPS, or 25 µg/ml NH_2-PS, COOH-PS or SiO_2 nanoparticles compared to untreated control cells. Commercial size exclusion chromatography columns were used to isolate the EVs. The results obtained after ANOVA analysis on all common proteins identified in the EV isolated from cells exposed to LPS or the different nanoparticles in respect to untreated cells are shown (see Methods for details) (Separate excel file)

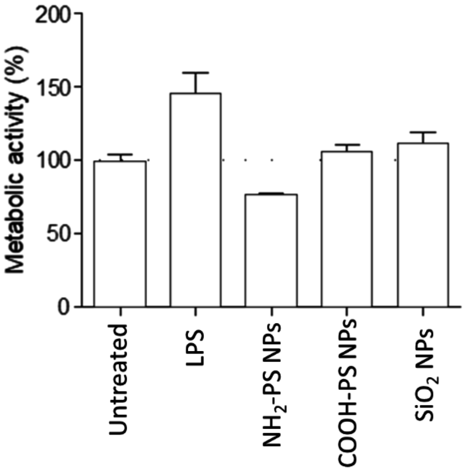

Supplementary Figure 1: Metabolic activity of MPI cells after exposure to nanoparticles. The metabolic activity of MPI cells was determined by means of an MTT assay after exposure for 1 hour to fresh medium (untreated cells), 100 ng/ml LPS, or 25 µg/ml nanoparticles (NH_2 PS, COOH PS or SiO_2 nanoparticles) and 23 hours of recovery in EV-depleted medium. The results are the mean and standard deviation over 3 replicates, normalised by the results in untreated cells. LPS treatment induced an increase in the cellular metabolic activity (146 ± 14% compared to 100 ± 4% for untreated cells). It is known that LPS can accelerate cell growth [3, 4]. NH_2-PS nanoparticles caused the metabolic activity to decrease to 77 ± 1 %. We have previously shown that subtoxic concentrations of NH_2-PS nanoparticles are able to affect mitochondrial functioning causing a mitochondrial hyperpolarization, which can explain the decrease of MTT conversion observed here [1]. COOH-PS NPs and SiO_2 NPs did not show substantial effects on cellular metabolic activity (106 ± 5 % and 112 ± 8 %, respectively).

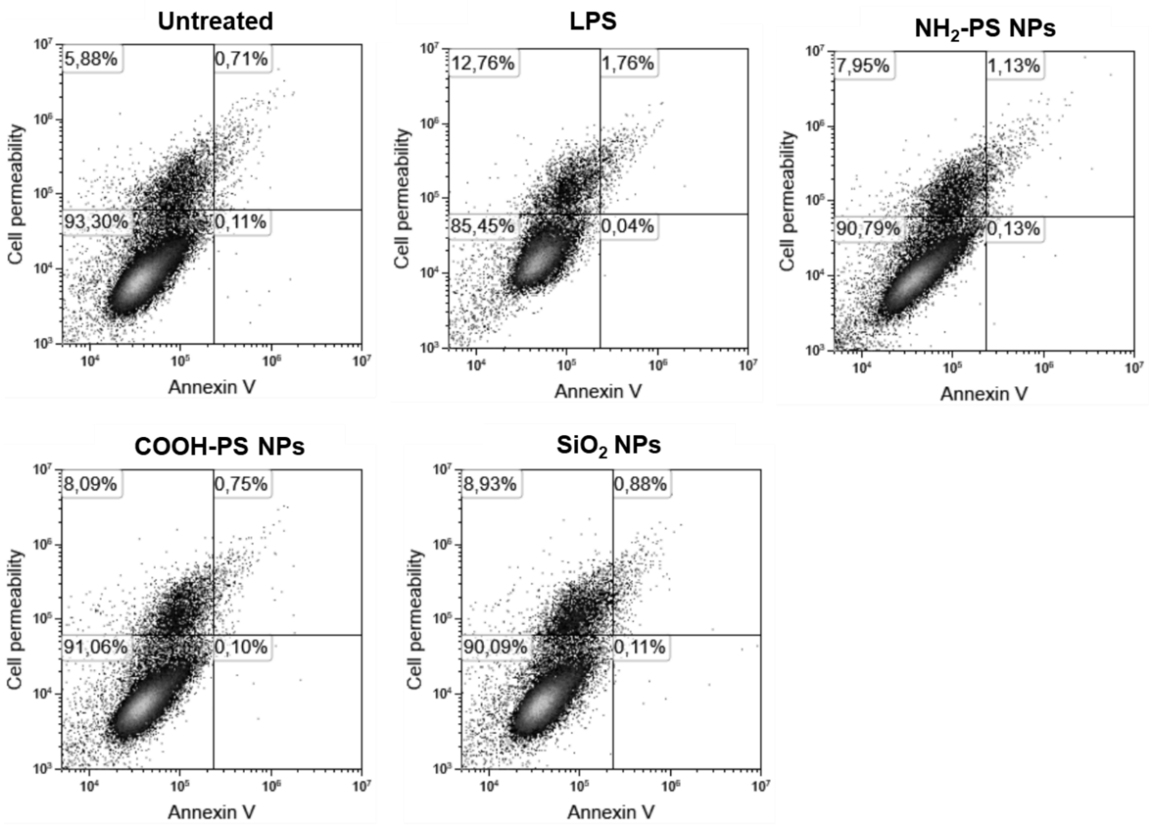

Supplementary Figure 2: Apoptotic response and cell death in MPI cells after exposure to nanoparticles. Annexin V – SYTOX staining was used to monitor eventual presence of apoptotic cells and changes in cell membrane permeability. Representative examples of Annexin V – SYTOX double scatter plots after exposure for 1 hour to fresh medium (untreated cells), 100 ng/ml LPS, or 25 µg/ml nanoparticles (NH_2 PS, COOH PS or SiO_2 nanoparticles) and 23 hours of recovery in EV-depleted medium. The percentage of intact cells (Annexin V negative and no increased cell permeability), apoptotic cells (Annexin V positive cells without increase in cell permeability), and necrotic cells (Annexin V positive cells with increased cell permeability) are shown. No evident signs of apoptosis and/or necrosis were found. Only 1-2% of the total cells were apoptotic (double positive Annexin V and cell permeability) for all conditions. Necrotic cells represented approximately 6.5% in the untreated condition, 14.5% after LPS exposure and 9-10% after exposure to nanoparticles. Given the minimal toxicity that was detected, these conditions were selected to limit the presence of cell debris or apoptotic bodies which could contaminate the isolated EVs, thus obscuring the results.

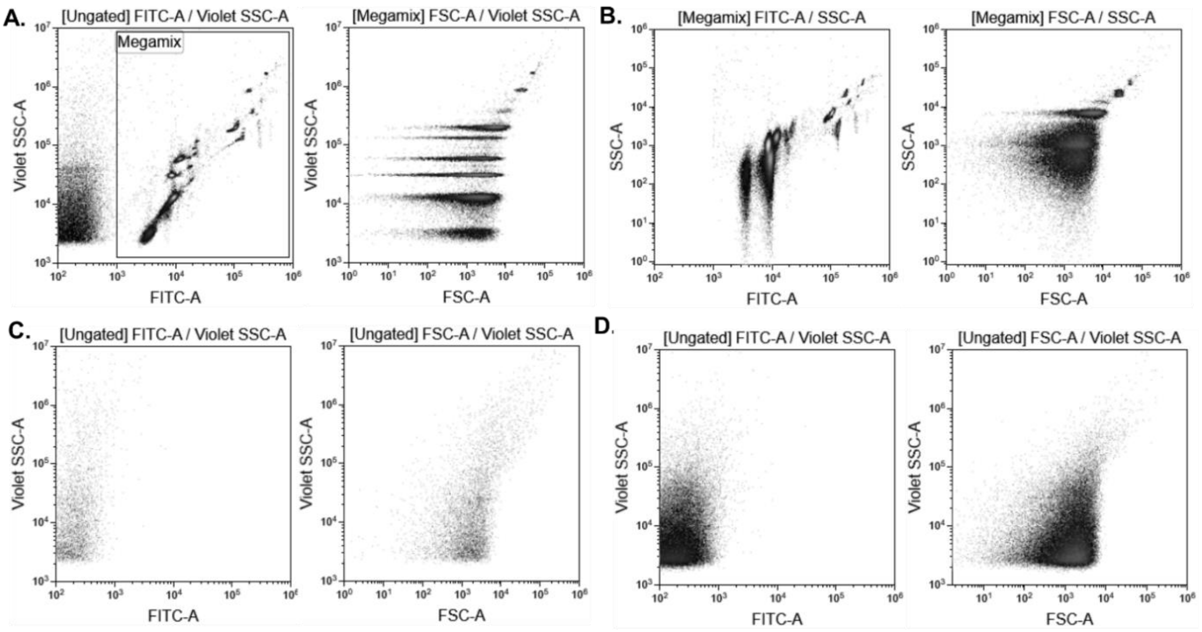

Supplementary Figure 3: High-sensitivity flow cytometry using CytoFlex. (A-D) Double scatter plots of (Violet) side scatter (SSC-A) *versus* the FITC-A fluorescence or forward scatter (FSC-A) are shown. **(A-B)** Recording of Megamix mixture (Megamix-Plus SSC and Megamix-Plus FSC mixed at a 1:1 ratio) which are fluorescently dyed beads with sizes between 100 nm and 900 nm. In panel A the results obtained using the 405 nm laser for side scattering detection are shown, while in B the same samples were measured using a standard 488 nm laser. Using the 405 nm violet side scatter (Violet SSC-A) allowed to achieve higher sensitivity in comparison to what is obtained with a regular 488 nm side scatter (SSC-A). **(C)** Background noise of the cytometer measured by running PBS for 2 minutes. **(D)** Representative example of the events recorded in a 2-minute measurement of a SEC fraction of EVs isolated from untreated MPI cells. Compared to PBS background (C) a clear increase in the recorded events is noted when measuring EVs .

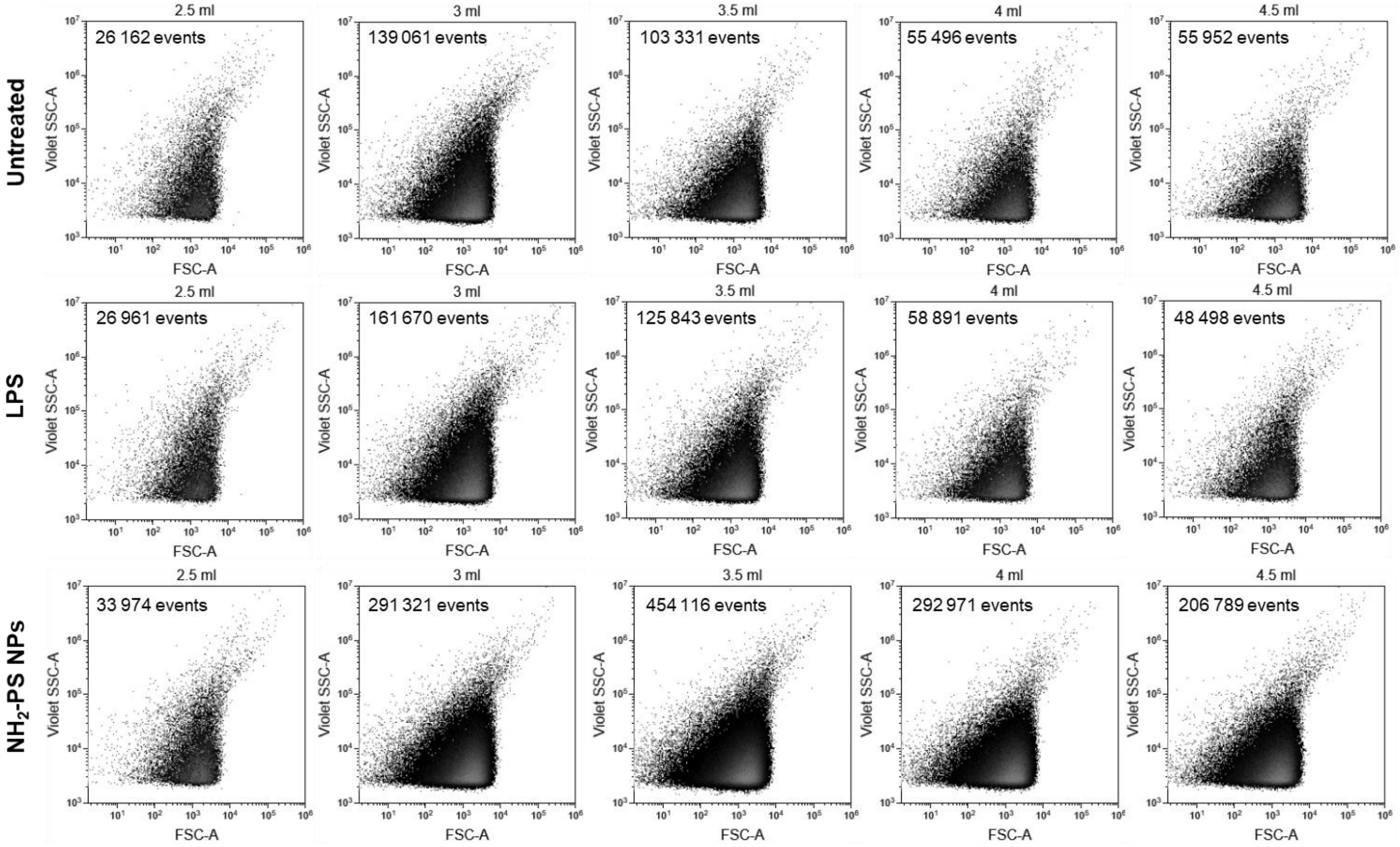

Supplementary Figure 4. High-sensitivity flow cytometric analysis of EVs using CytoFlex. In-house made size exclusion chromatography columns were used to isolate the EVs. Flow cytometric analysis of EVs collected after exposure for 1 hour to fresh medium (untreated cells), 100 ng/ml LPS, or 25 µg/ml NH_2 PS and 23 hours of recovery in EV-depleted medium. EV fractions corresponding to an elution volume between 2.5 and 4.5 ml were measured for 2 minutes

and the corresponding double scatter plots of violet side scatter (Violet SSC-A) *versus* forward scatter (FSC-A) are shown. For each recorded fraction, the corresponding total number of events is plotted in Figure 3 (bar plots).

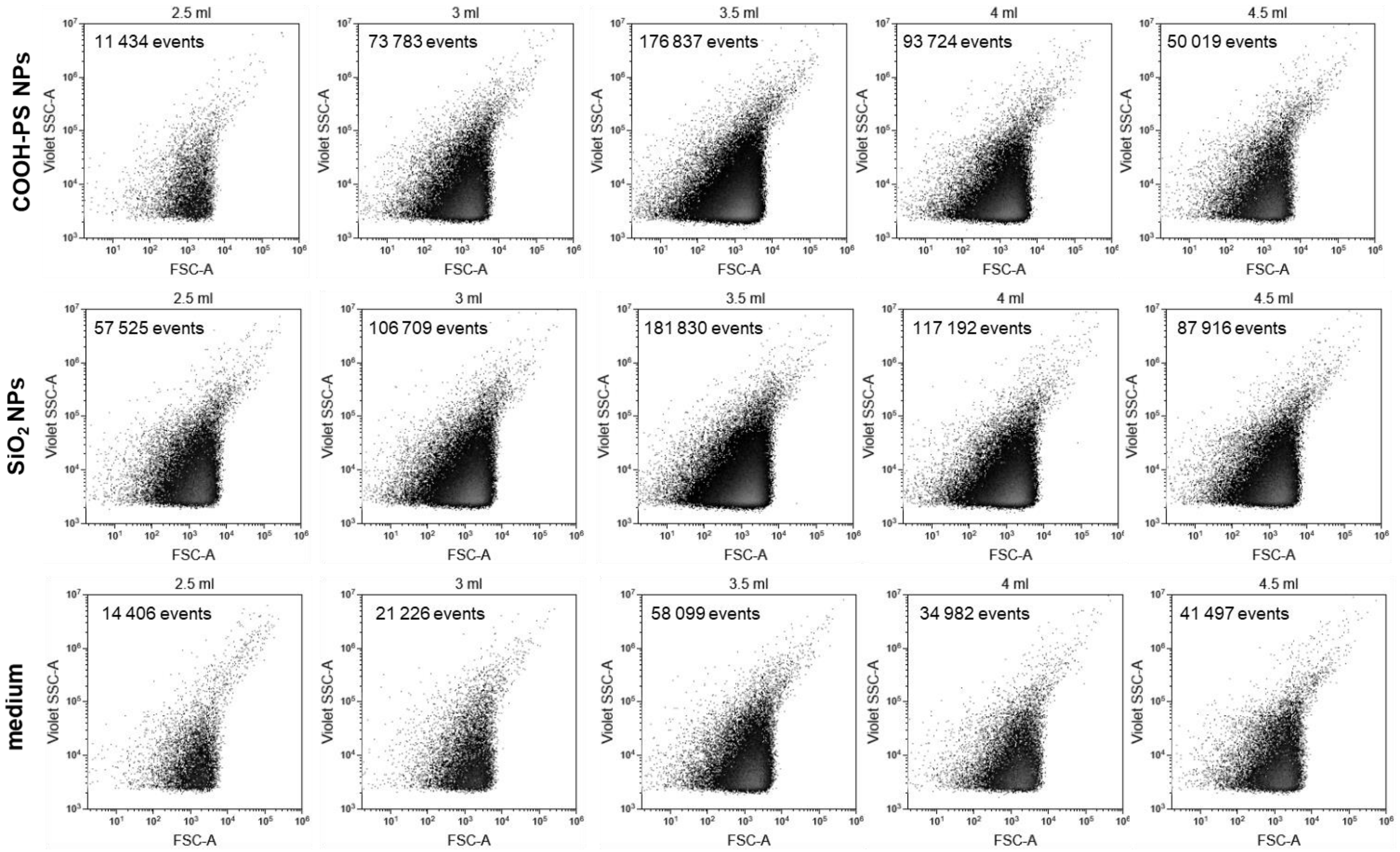

Supplementary Figure 5. High-sensitivity flow cytometric analysis of EVs using CytoFlex. In-house made size exclusion chromatography columns were used to isolate the EVs. Flow cytometric analysis of EVs collected after exposure for 1 hour to 25 µg/ml COOH PS nanoparticles and SiO_2 nanoparticles and 23

hours of recovery in EV-depleted medium. EV-depleted medium (medium) was processed in similar way and served as a control. EV fractions corresponding with an elution volume between 2.5 and 4.5 ml were measured for two minutes and the corresponding double scatter plots of violet side scatter (Violet SSC-A) *versus* forward scatter (FSC-A) are shown. For each recorded fraction, the corresponding total number of events is plotted in Figure 3 (bar plots).

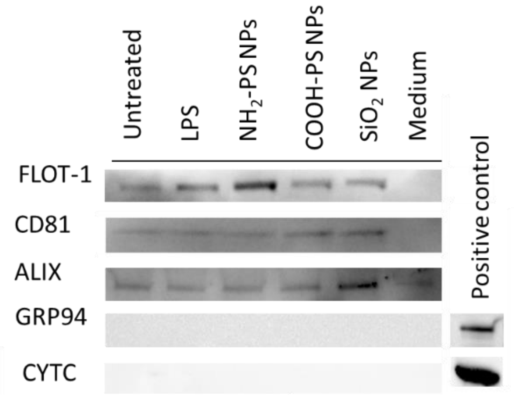

Supplementary Figure 6: Western blot of EVs produced by MPI cells after exposure to nanoparticles. Commercial size exclusion chromatography columns were used to isolate the EVs. EV fractions corresponding with an elution volume between 3 and 4 ml were pooled, and equal volumes were loaded. Detection of flotillin-1 (FLOT-1), CD81, ALG-2 interacting protein X (ALIX), GRP94 and cytochrome C (CYTC) are shown. For GRP94 and CYTC, 10 µg cell lysate of MPI cells was loaded as a positive control. Membrane-bound FLOT-1 and CD81, and cytosolic ALIX are proteins usually enriched in EVs, whereas the endoplasmic reticulum chaperone GRP94 and the mitochondrial CYTC are cell-derived proteins not expected in the isolated EVs. These combined results confirmed separation of EVs in the recovered fractions and absence of cellular contamination.

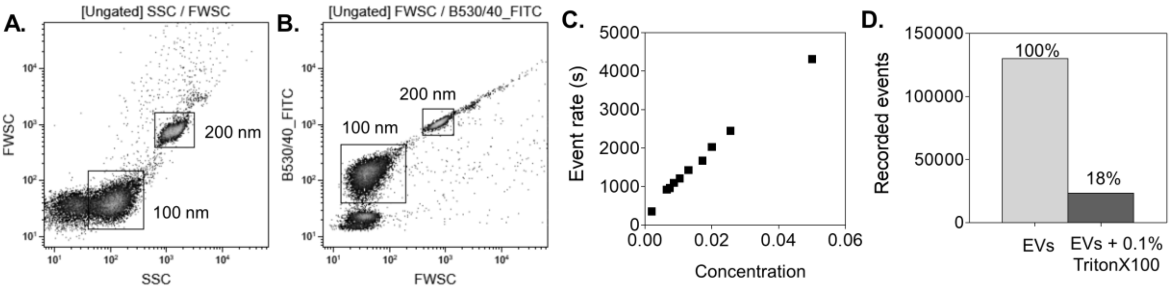

Supplementary Figure 7: High-sensitivity flow cytometry using BD Influx instrument. (A-B) Sensitivity of fluorescence triggering detection was illustrated by 100 nm and 200 nm fluorescently dyed beads. The double scatterplots for the forward scatter (FWSC) *versus* the side scatter (SSC), and the fluorescence (BP530/40) *versus* FWSC show the resolution between the two bead populations. **(C)** The absence of detector swarming validated by a serial dilution of a CFDA-SE stained EV sample isolated using commercial size exclusion chromatography. The results demonstrate good performance of the BD Influx instrument during concentration measurements. **(D)** The addition of the detergent Triton-X100 (0.1% v/v) for 15 minutes at room temperature to CFDA-SE stained EVs results in a remarkable decrease in EV number, which confirms the vesicular nature of the recorded EVs.

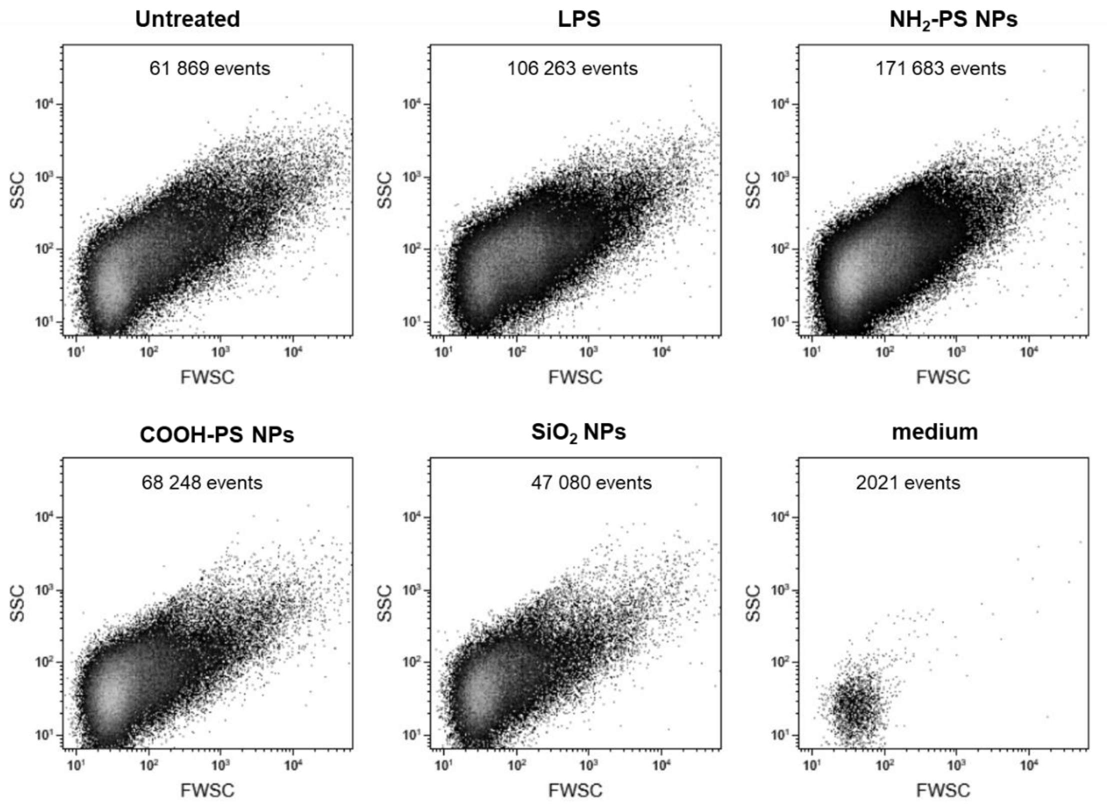

Supplementary Figure 8: Scatter plots of CFDA-SE stained EVs produced by MPI cells after exposure to LPS, NH_2-PS nanoparticles, COOH-PS nanoparticles, or SiO_2 nanoparticles, or left without treatment. Commercial size exclusion chromatography columns were used to isolate the EVs. Side scatter (SSC) and forward scatter (FWSC) signals of the CFDA-SE positive events from a representative experiment, obtained by high-sensitivity flow cytometry using a BD Influx instrument are shown.

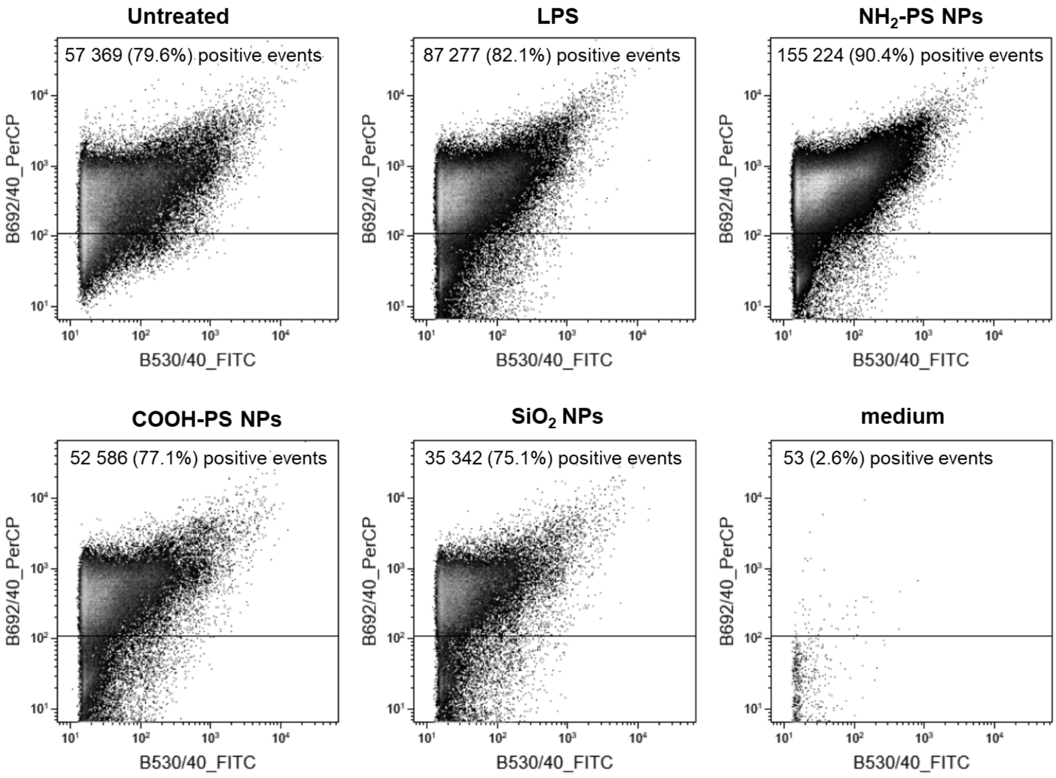

Supplementary Figure 9: Fluorescence plots of double CFDA-SE and CD9-BrilliantBlue700 stained EVs produced by MPI cells after exposure to LPS, NH_2-PS nanoparticles, COOH-PS nanoparticles, or SiO_2 nanoparticles, or left without treatment. Commercial size exclusion chromatography columns were used to isolate the EVs. Double CD9-BB700 (BP692/40) and CFDA-SE (BP530/40) stained EVs were analysed by high-sensitivity flow cytometry using a BD Influx instrument. The number and percentage of CD9-positive events is shown from one representative experiment.

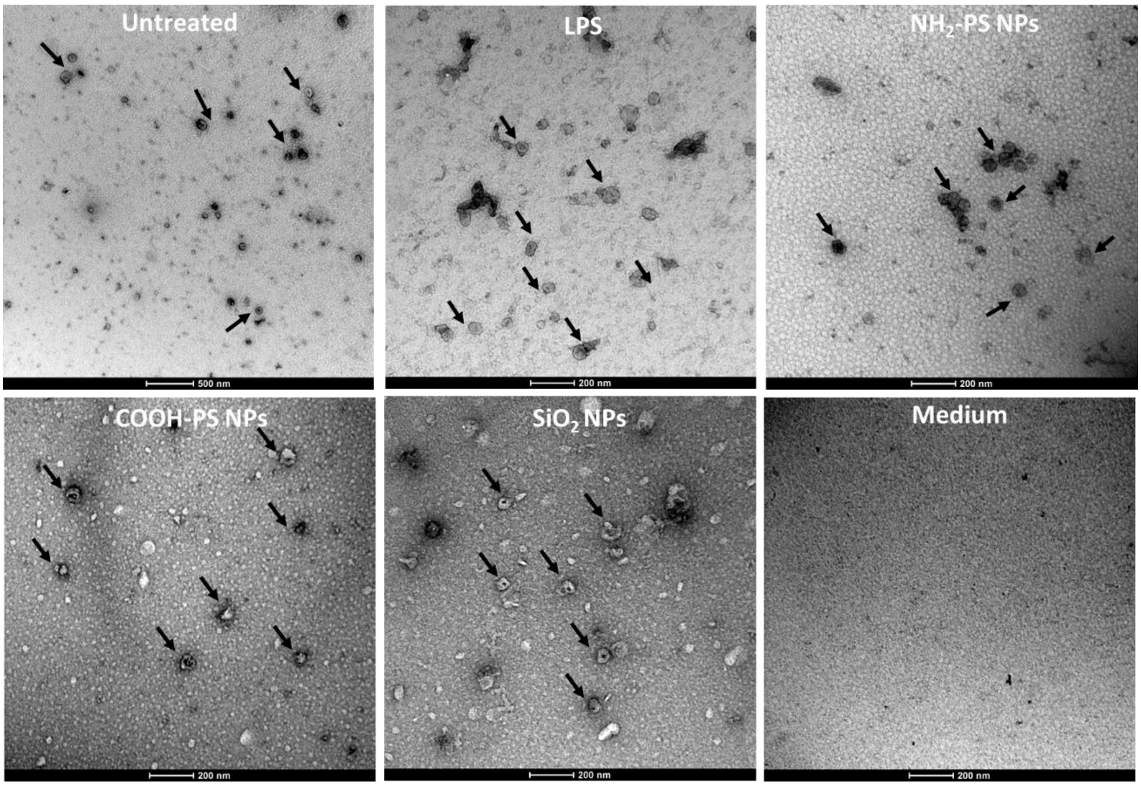

Supplementary Figure 10: Characterisation of EV morphology from MPI cells after exposure to LPS, NH_2-PS nanoparticles, COOH-PS nanoparticles, or SiO_2 nanoparticles, or left without treatment. Transmission electron microscopy was performed on pooled SEC fractions corresponding to an elution volume between 3 and 4 ml. Commercial size exclusion chromatography columns were used to isolate the EVs. Untreated cells (untreated) and cell culture medium without cells subjected to the same isolation procedure (medium) were used as controls. Scale bars: 200 nm. The arrows indicate examples of EVs in the samples.

References

1. Deville, S., et al., *Time-resolved characterization of the mechanisms of toxicity induced by silica and amino-modified polystyrene on alveolar-like macrophages.* Archives of toxicology, 2019: p. 10.1007/s00204-019-02604-5.
2. Vasse, G.F., et al., *Collagen morphology influences macrophage shape and marker expression in vitro.* Journal of Immunology and Regenerative Medicine, 2018. **1**: p. 13-20.
3. Lin, T.-Y., et al., *Lipopolysaccharide-promoted proliferation of Caco-2 cells is mediated by c-Src induction and ERK activation.* BioMedicine, 2015. **5**(1): p. 5.
4. Hattar, K., et al., *Endotoxin induces proliferation of NSCLC in vitro and in vivo: role of COX-2 and EGFR activation.* Cancer Immunology, Immunotherapy, 2013. **62**(2): p. 309-320.
